# A *NHEJ1* mutator allele influencing germline mutation rates sex-specifically in humans

**DOI:** 10.1126/sciadv.aed2571

**Published:** 2026-07-31

**Authors:** Kun Wu, Jiuhong Nan, Haoxuan Liu

**Affiliations:** ^1^ Center for Evolutionary & Organismal Biology, Department of Pharmacy, Center for Regeneration and Aging Medicine, the Fourth Affiliated Hospital of School of Medicine, International School of Medicine, and International Institutes of Medicine, Zhejiang University School of Medicine, Hangzhou 310058, China.; ^2^ Liangzhu Laboratory, Zhejiang University School of Medicine, Hangzhou 311121, China.; ^3^ Zhejiang-Denmark Joint Laboratory of Regeneration and Aging Medicine, Yiwu 322000, China.

## Abstract

Germline mutations play a pivotal role in evolution and are the primary cause of hereditary diseases in humans. Although interpopulation and interspecific variations in the mutation rate and spectrum are observed, their underlying genetic basis is still unclear. In this study, we explore the genetic regulation of germline mutation rates using one of the largest publicly available datasets derived from parent-offspring whole-genome sequencing. We first showed that germline mutation rates are strongly correlated between siblings, suggesting the influence of heritable factors. We then performed a genome-wide association study (GWAS), identifying 14 loci significantly associated with mutation rates, Notably, a lead single-nucleotide polymorphism (SNP) in the *NHEJ1* gene, critical for DNA repair, was linked to a 27% increase in maternal mutation rates and a significant shift toward A-to-T mutations only in females. This mutator allele is particularly prevalent in East Asian populations, where a similar shift in the A-to-T mutation spectrum has been observed. Evolutionary analysis suggests that this allele likely emerged in the common ancestor of humans and the *Pan* genus. Furthermore, the genotype at the lead SNP position in *Pan* species is also the same as the human mutator allele, supporting the potential role for this allele to explain the accelerated A-to-T mutations observed in the human-*Pan* lineage. Together, these results indicate that genetic variants, particularly in DNA repair pathways, contribute to variation in mutation rates and spectra across individuals and populations, with a notable sex-specific effect.

## Teaser

GWAS reveals a DNA repair variant in *NHEJ1* that increases maternal germline mutation rate and drives an A-to-T mutational shift.

## INTRODUCTION

Germline mutations are fundamental to evolution and, at the same time, represent the primary cause of thousands of hereditary diseases in humans (*1*, *2*). The germline mutation rate, defined as the number of new mutations per site per generation, not only responds to natural selection but also evolves as a quantitative trait (*3*–*5*). Moreover, both the rate and the spectrum of germline mutations constitute one of the major forces shaping the human genome (*6*–*8*). Understanding the genetic regulation of this process is therefore a central question in evolutionary genetics. Because most mutations are deleterious, natural selection has suppressed the mutation rate to a relatively low level (*4*). With the advent of whole-genome sequencing, de novo germline mutation rates have now been directly estimated across hundreds of species (*9*). In humans, this rate is ∼1.1 × 10^−8^ per site per generation (*10*).

At the molecular level, the germline mutation rate is determined by the coordinated activity of hundreds of genes involved in DNA replication, proofreading, and repair (*11*, *12*). Any mutation that alters the function or expression of these genes has the potential to change the mutation rate (*13*). Mutations that severely compromise these pathways often give rise to hypermutator phenotypes, which lead to severe developmental disorders and are rapidly eliminated by selection (*14*). In contrast, weak modifiers of the mutation rate may persist and drift within populations for several reasons. First, natural selection acting on the mutation rate is indirect, also known as second-order selection, because it operates only through the fitness consequences of new mutations (*15*–*17*). This form of selection is much weaker than direct, or first-order, selection, and in sexually reproducing populations recombination further reduces its efficiency (*18*). Second, because the human mutation rate is already extremely low, the fitness effects of small modifiers are correspondingly negligible (*s* < 1/*N*_e_) (*4*, *18*). Third, the relatively small effective population size of humans renders such weak modifiers effectively invisible to natural selection (*4*).

Consistent with this reasoning, accumulating evidence indicates that the human germline mutation rate has recently evolved and differs across populations (*6*, *7*). Using segregating single-nucleotide polymorphisms (SNPs) as proxies for germline mutations, Harris (*7*) first demonstrated significant differences in mutational spectra among human populations, with certain mutation types elevated in specific groups. More recent analyses of de novo mutations in sequenced family trios confirmed that both mutation rate and spectrum vary among populations (*19*). These findings strongly suggest that genetic modifiers of the germline mutation rate differ between populations, although specific modifiers remain largely unknown. Moreover, even within the same population, individuals vary substantially in their mutation counts, ranging from ∼25 to 110 de novo mutations per generation even after accounting for parental age differences (*20*–*22*). Whether this variation reflects purely stochastic processes or has a genetic basis remains an unresolved question.

In this study, we address this problem using one of the largest publicly available germline mutation datasets derived from parent-offspring whole-genome sequencing (*23*). We first test whether genetic factors contribute to mutation rate variation by evaluating the distribution of mutation counts. We find that the observed distribution is significantly more dispersed than expected under a Poisson process and that siblings display significant correlation in mutation number, both patterns consistent with the presence of heritable modifiers. To identify such modifiers, we performed genome-wide association analyses linking parental genotypes to offspring mutation counts (fig. S1). We detected 12 SNPs of paternal origin and 22 SNPs of maternal origin that are significantly associated with mutation rate. Functional annotation suggests that 80% of these loci have the potential to regulate gene expression. One lead maternal SNP is located in the intronic region of *NHEJ1*, a gene essential for the nonhomologous end joining (NHEJ) repair pathway. Although this variant shows no detectable effect on mutation rate or spectrum in males, females carrying the allele exhibit a 27% increase in germline mutation rate as well as a significant shift toward A-to-T mutations. Together, these findings provide new insights into the genetic regulation of germline mutation rate and highlight the role for sex-specific genetic modifiers.

## RESULTS

### Evidence that the germline mutation rate is influenced by genetic factors

We first asked whether germline mutation rate variation shows evidence of genetic control. The dataset obtained from Werling *et al.* (*24*) and An *et al.* (*23*) contained 1902 families, each consisting of a father, a mother, and two children. The number of germline mutations in the two children of each family was determined through parent-offspring whole-genome sequencing. Because mutation number is strongly correlated with parental age at conception (*20*, *25*, *26*), we first corrected the mutation counts in each family for paternal and maternal age using a linear model (fig. S2; Methods).

**Fig. 1.**
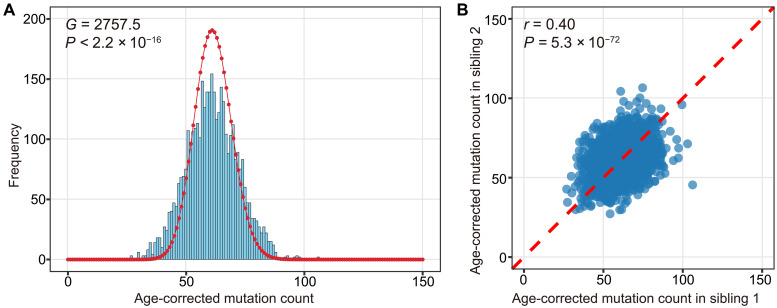
Distribution of age-corrected de novo mutation counts in quartet families. (**A**) Histogram of observed mutation counts after adjusting for parental ages in offspring (blue bars) with the expected values from a Poisson distribution overlaid as a red curve. (**B**) Scatterplot shows the correlation of mutation counts between the two siblings within each family.

If de novo mutations occur independently at a constant rate, the number of mutations per individual should follow a Poisson distribution (*27*). However, the presence of genetic modifiers in the population would increase the variance, producing a distribution that is more dispersed than expected under the Poisson model. After age correction, the number of mutations across the 1902 families was significantly more dispersed than a Poisson distribution with a mean of 61.4 (Fig. 1A; likelihood ratio test, *G* = 2757.5, *P* ≈ 0).

Furthermore, if heritable modifiers exist, siblings within the same family would be more likely to have similar mutation counts. Consistent with this prediction, the Pearson correlation coefficient for age-corrected mutation counts between siblings across the 1902 families was 0.40 (Fig. 1B; *r* = 0.40, *P* = 5.3 × 10^−72^), which is significantly higher than randomly shuffled sibling pairs (*P* < 0.0001; fig. S3). These two observations are consistent with the presence of heritable modifiers of germline mutation rate. Although environment factors could contribute to these patterns, the high sibling correlation strongly implicates genetic factors.

### GWAS analysis identifies 14 genome-wide significant loci

To map potential genetic modifiers, we performed a genome-wide association study (GWAS) to identify loci influencing mutation rates. In this analysis, each family contributed a single data point, and the phenotype of interest was defined as the average mutation count across the two siblings after adjusting for parental age. Unlike most GWAS designs in which phenotype and genotype are measured in the same individuals, the number of germline mutations in the offspring is determined by mutations that occurred in the paternal sperm and maternal egg at the time of fertilization. For this reason, we carried out two separate analyses: one that related offspring mutation counts to paternal genotypes and another that related them to maternal genotypes (fig. S1).

After genomic data acquisition, genotype imputation, minor allele frequency filtering, and quality control, GWAS analyses were conducted on paternal (*n* = 1606; ages 16 to 58 years; mean age = 33 years) and maternal (*n* = 1616; ages 17 to 47 years; mean age = 31 years) datasets. Ancestry inference confirmed that this population is of European descent (Methods). The full analyses tested 6,187,686 SNPs in the paternal dataset and 6,356,221 SNPs in the maternal dataset. The numbers of independent SNPs (120,224 and 122,213, respectively, defined by *r*^2^ < 0.1; Methods) were used to determine Bonferroni-corrected significance thresholds. Quantile-quantile plots showed no evidence of inflation (fig. S4; λ_father = 0.99; λ_mother = 0.98), indicating well-calibrated test statistics and no evidence of population stratification or systematic bias.

**Fig. 2.**
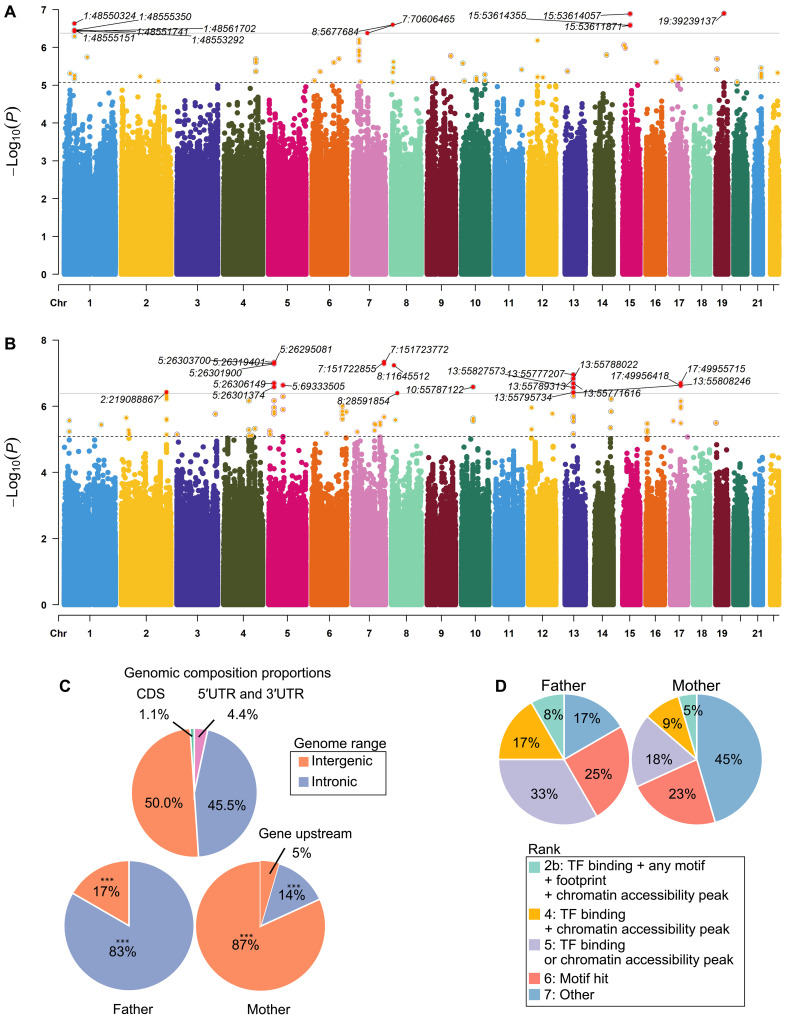
Results of SNP-based meta-analysis for germline mutation rate. Manhattan plots for the paternal (**A**) and maternal (**B**) GWAS analyses. The *y* axis indicates the negative log_10_-transformed *P* values of SNP associations, whereas the *x* axis corresponds to genomic positions along the chromosomes. Solid and dashed lines indicate the genome-wide significance threshold (*P* = 0.05/*N*; *N* denotes the number of completely independent loci; see Methods for details) and the suggestive association threshold (*P* = 1/*N*), respectively. Red dots denote top SNPs with *P* values below the significance threshold, labeled with their genomic coordinates (SNP ID shown as chromosome:position). (**C**) Functional annotation based on genomic loci for the 34 significant top SNPs. The significance of the proportions of intergenic and intronic regions in the paternal and maternal lineages was evaluated using a permutation test based on the genomic composition of GRCh38 (version 46), with 10,000 resampling iterations. ***P < 0.001. CDS, coding sequence; 3′UTR, 3′ untranslated region; 5′UTR, 5′ untranslated region. (**D**) Transcriptional potential of the 34 significant top SNPs, with data sourced from RegulomeDB. TF, transcription factor.

**Fig. 3.**
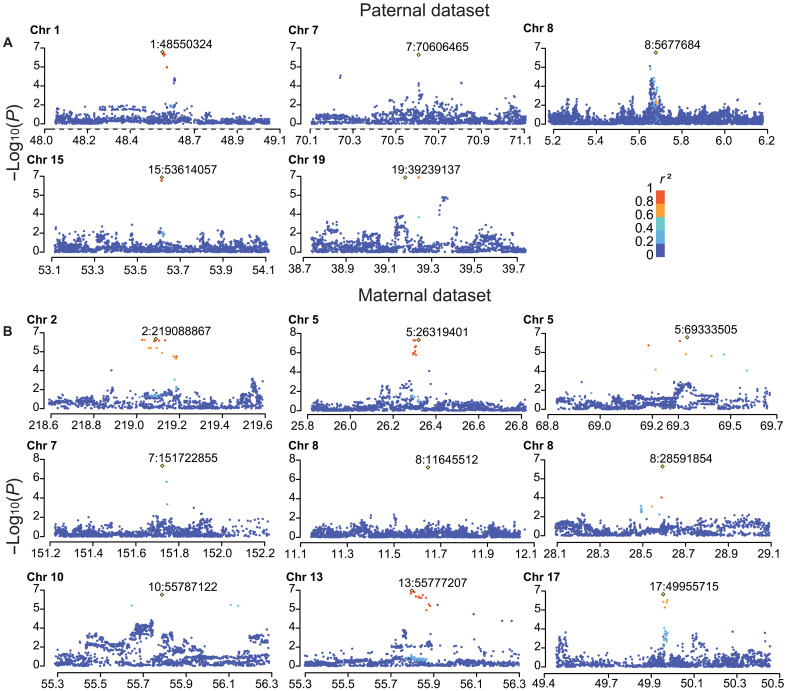
LD plot for the local region. Regional LD plot for the 14 lead SNPs in the paternal (**A**) and maternal (**B**) datasets. The *x* and *y* axes represent the chromosomal position and the negative log_10_-transformed *P* values of SNPs, respectively. Yellow diamonds denote the lead SNP (with the lowest *P* value in the region). The *r*^2^ values indicate the LD between other SNPs and the lead SNP.

In total, we identified 34 SNPs that surpassed the genome-wide significance threshold (12 paternal and 22 maternal; Fig. 2, A and B, and additional files 1 and 2), among which 14 were independent lead SNPs (5 paternal and 9 maternal; Figs. 2 and 3 and Table 1). None of the significant loci overlapped between the paternal and maternal datasets, implying sex-specific effects (Fig. 2, A and B). In the paternal analysis, most significant SNPs (83%) were located in intronic regions (Fig. 2C), and most of these (83%) showed evidence of potential transcriptional activity (Fig. 2D). In the maternal analysis, most significant SNPs (87%) were intergenic (Fig. 2C), although more than half (55%) overlapped with regions of active chromatin (Fig. 2D). Together, these findings indicate that most of the identified loci likely exert their effects through the regulation of gene expression.

**Table 1.** Genomic loci of lead SNPs associated with mutation rate in the GWAS. SNP ID: genomic coordinate of the lead SNP (chromosome:position); Locus: cytogenetic band, GRCh38 build; *P*: *P* value from the GWAS analysis.

SNP ID	Locus	Mutation type	Annotation	*P*
Maternal background				
2:219088867	2q35	C>T	*NHEJ1*, intronic	3.72 × 10^−7^
5:26319401	5p14.1	C>T	Intergenic	4.68 × 10^−8^
5:69333505	5q13.2	T>A	*CCDC125*, upstream	2.31 × 10^−7^
7:151722855	7q36.1	C>T	*PRKAG2*, intronic	4.58 × 10^−8^
8:11645512	8p23.1	A>G	Intergenic	5.77 × 10^−8^
8:28591854	8p21.1	G>A	Intergenic	4.02 × 10^−7^
10:55787122	10q21.1	T>A	Intergenic	2.63 × 10^−7^
13:55777207	13q21.1	C>T	Intergenic	1.10 × 10^−7^
17:49955715	17q21.33	C>T	Intergenic	2.00 × 10^−7^
Paternal background				
19:39239137	19q13.2	C>T	Intergenic	1.26 × 10^−7^
15:53614057	15q21.3	A>G	*WDR72*, intronic	1.29 × 10^−7^
1:48550324	1p33	C>T	*AGBL4*, intronic	2.34 × 10^−7^
8:5677684	8p23.2	G>C	Intergenic	2.50 × 10^−7^
7:70606465	7q11.22	C>G	*AUTS2*, intronic	4.16 × 10^−7^

We additionally estimated SNP-based heritability using a mixed linear model [restricted maximum likelihood (REML)] framework that partitions phenotypic variance into an additive genetic component captured by the genomic relationship matrix (*V*_g_) and a residual component *V*_e_, with ℎ^2^ = *V*_g_/(*V*_g_ + *V*_e_). This analysis yielded consistent estimates for the paternal and maternal components (ℎ^2^ ≈ 0.38 in both cases), indicating that ∼38% of the variation in mutation rate is attributable to additive genetic variation tagged by common SNPs.

### *NHEJ1* as a sex-specific germline mutation rate modifier

The lead SNPs mapped to six genic regions (Table 1; *WDR72*, *AGBL4*, *AUTS2*, *NHEJ1*, *CCDC125*, and *PRKAG2*). Of these, *WDR72*, *AGBL4*, and *AUTS2* are known prognostic markers in cancer (*28*, *29*), suggesting possible roles in regulating mutation processes. *NHEJ1* emerged as a strong candidate because of its well-established role in DNA repair. *NHEJ1* encodes a key factor in the NHEJ pathway by promoting the ligation of mismatched and noncohesive DNA ends (*30*–*32*). NHEJ is the predominant double-strand break repair pathway in human cells and is responsible for ∼80% of all such repairs (*33*).

**Fig. 4.**
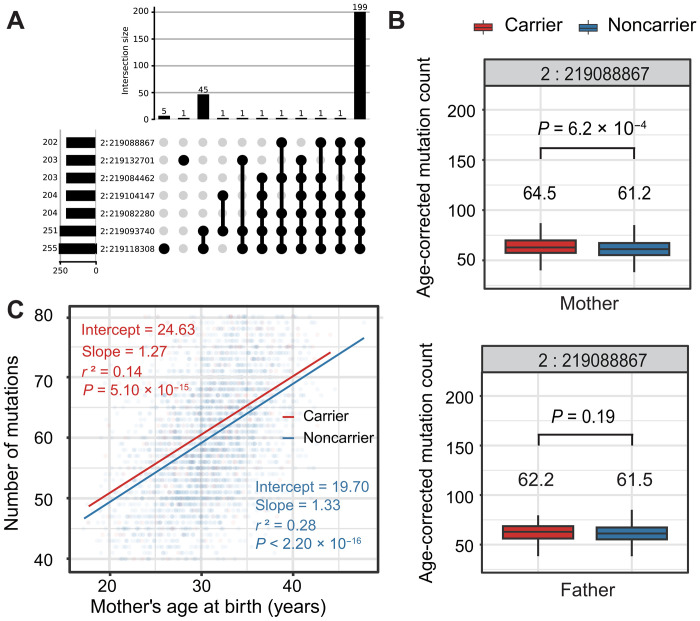
Functional analysis of the lead SNP in *NHEJ1*. (**A**) Intersection plot of suggestively associated SNPs in the maternal dataset. The bars in the top section show the number of SNPs shared in each specific combination of samples represented below. The black circles and connecting lines indicate which samples are included in each intersection. The side bars on the left depict the total number of samples in which each SNP is present. (**B**) Contribution of the lead SNP in *NHEJ1* to mutation rate. Age-corrected mutation count in samples carrying versus not carrying the lead SNP (2:219088867) in the maternal (top) and paternal (bottom) datasets. (**C**) Correlation between the age and the number of mutations in individuals carrying and not carrying the lead SNP in the maternal dataset.

Within *NHEJ1*, we identified a lead SNP in the maternal dataset (2:219088867) surrounded by six additional suggestively significant SNPs (Fig. 3 and table S1). This clustering is expected for true GWAS signals due to linkage disequilibrium (LD). Consistent with this, the lead SNP was present in 202 of 1616 mothers, and in 199 of these carriers (99%), the surrounding six SNPs were also linked (Fig. 4A). Functional annotation indicated that six of the seven SNPs had regulatory potential (table S1). To assess the impact of this lead SNP on the mutation rate, we compared mutation rates between carriers and noncarriers of this lead SNP in both the maternal and paternal datasets. In the maternal dataset, carriers of the lead SNP exhibited a significant increase in mutation rate (Fig. 4B, *n*_carrier = 202, *n*_noncarrier = 1414; Wilcoxon test, *P* = 0.0006), whereas no such effect was observed in the paternal dataset (Fig. 4B, *n*_carrier = 158, *n*_noncarrier = 1448; *P* = 0.19). On average, mothers who carried this SNP passed down 3.3 more mutations per generation than noncarriers. Given that maternal-origin mutations typically account for only about 20% of all germline mutations, we estimate that noncarrier mothers pass down 12.24 mutations per generation (Methods). Because this SNP affects only the maternal mutation rate, we conclude that it is associated with a 27% increase (3.3/12.24) in the maternal germline mutation rate (Fig. 4B).

To assess whether this SNP influences the rate of mutation accumulation with age, we calculated the correlation between age at conception and the number of germline mutations in the offspring for carriers and noncarriers within the maternal dataset. The slopes of the linear regression for both groups were nearly identical (Fig. 4C; *t* test, *P* = 0.62; Methods), suggesting that mutations accumulate at a similar rate with age in both carriers and noncarriers. Such a pattern is unlikely to be the result of spurious SNPs identified in the GWAS, further supporting the reliability of our analysis. In addition, the intercept for the carrier was significantly higher than that of the noncarrier (*t* test, estimate = −3.10, *P* = 1.28 × 10^−7^; Methods). This provides parallel evidence that, at the same maternal age, the carrier has, on average, ∼3 more mutations than the noncarrier. Such a parallel pattern also indicates that the SNP may influence mutation rates during development before sexual maturity. In contrast, neither the slope nor the intercept differs between paternal carriers and noncarriers (*t* tests, both *P* > 0.5; fig. S5).

### Evolutionary history of the *NHEJ1* mutator allele and its contribution to natural variation in the mutation spectrum

**Fig. 5.**
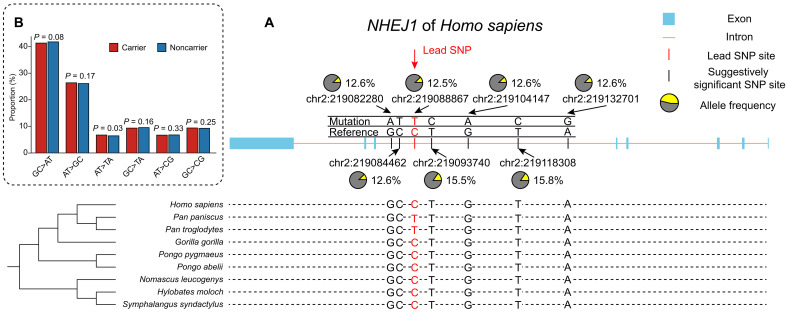
Evolutionary history of the *NHEJ1* mutator allele. (**A**) Allele frequency and distribution of lead and suggestively associated *NHEJ1* SNPs in humans and other primates. The pie chart represents the allele frequencies of this SNP in the dataset we analyzed. The phylogenetic tree was constructed on the basis of *NHEJ1* gene sequences using IQ-TREE (*49*), and its topology is consistent with the species tree based on TimeTree (*50*). (**B**) Comparison of mutational spectra of individuals carrying and not carrying the lead SNP in the maternal dataset. The *P* values shown above were calculated using a permutation test. Only the lead SNP and six surrounding SNPs are analyzed here.

To trace the origin of the *NHEJ1* mutator allele, we first examined its allele frequency in modern human populations using gnomAD (v4.1.0) (*34*). The allele occurs at an overall frequency of ∼0.06, indicating that it is a minor allele in humans. We next investigated the ancestral state by examining the ape superfamily. We acquired the reference genomes of eight primate species and constructed a phylogenetic tree using the *NHEJ1* gene sequence (Fig. 5A). The resulting topology is consistent with the species tree. At the position of the lead SNP, the closest genera to humans, including *Pan paniscus* and *Pan troglodytes*, carry the genotype “T,” which is the same as the mutator allele “T” found in humans. In contrast, all other great apes and gibbons carry the “C” allele, which corresponds to the human major allele “C.” This pattern suggests that the ancestral state of this site in the ape superfamily is “C,” with the “T” allele emerging in the common ancestor of humans and the *Pan* genus. Consequently, the “T” allele likely became fixed in the *Pan* species while remaining polymorphic in humans.

Following population-genetic theory, the selective disadvantage of a mutator allele can be approximated as 2*sU* (*4*), where *s* is the mean selection coefficient against a new deleterious mutation and *U* is the mutator-induced increase in the deleterious mutation rate. Using 3.3 additional maternal mutations per generation, a coding fraction of ∼1.1%, ∼70% nonsynonymous among coding point mutations (*35*), and ∼70% deleterious among nonsynonymous mutations (*36*), we estimate *U* ≈ 0.02, which yields *N*_e_ (2*sU*) ≈ 0.4 for s ≈ 10^−3^ (*37*) and *N*_e_ ≈ 10^4^ (*38*), placing the allele near the nearly neutral boundary, so it could be nearly neutral or only weakly deleterious under these assumptions. This is compatible with the possibility that it is drifting within human populations.

A previous study examining the evolution of the human mutational spectrum has identified elevated A-to-T mutation rates across multiple evolutionary scales (*6*). In population comparisons, the A-to-T mutation rate was found to be significantly higher in East Asian populations relative to others. Similarly, interspecific comparisons revealed that the A-to-T mutation rate is elevated in the lineage shared by humans and the *Pan* genus (*6*). These findings suggest the presence of a genetic modifier influencing the mutation rate of this type.

To determine whether the mutator allele we identified contributes to this variation, we compared the relative proportions of the six mutation types between carriers and noncarriers within the maternal dataset. Among the six mutation types, only the proportion of A-to-T mutations showed a significant increase in carriers (Fig. 5B). Consistent with this, we found that the frequency of this allele is highest in the East Asian population [0.46, data from gnomAD (*34*)], potentially explaining the previously observed population differences. Furthermore, the genotype at the lead SNP position in the *Pan* genus is the same as the human mutator allele. Although we are unable to directly assess its effects in *Pan*, nonetheless, if the mutator allele exerts a similar effect in this lineage as it does in humans, the nucleotide variation at this position could explain the elevated A-to-T mutation rate observed in the shared lineage of humans and *Pan* (*6*).

## DISCUSSION

In this study, we provide strong evidence that genetic factors influence the germline mutation rate in humans. Our GWAS identified multiple loci that are significantly associated with mutation rates in both paternal and maternal datasets. Notably, a lead SNP in the *NHEJ1* gene, a critical player in the NHEJ DNA repair pathway, was associated with a significant increase in the maternal germline mutation rate. This suggests that genetic variants involved in DNA repair can modulate the mutation rate, potentially contributing to variation in mutation rates across populations and individuals. These findings open new avenues for understanding the genetic regulation of mutation rates and highlight the potential for sex-specific genetic modifiers.

Our findings also contribute to understanding how genetic variation, particularly in the *NHEJ1* gene, influences the mutation spectrum across populations and species. The *NHEJ1* mutator allele is significantly associated with a higher A-to-T mutation rate (Fig. 5B). This allele is most prevalent in East Asian populations, where a notable shift in the A-to-T mutation spectrum has been reported (*6*). This observation suggests that *NHEJ1* plays a key role in shaping the mutation spectrum at the population level. Furthermore, the *NHEJ1* mutator allele may help explain the accelerated A-to-T mutations observed in the human-*Pan* lineage (*6*). In *Pan* species, the genotype at the lead SNP position in *NHEJ1* is also the same as the human mutator allele. Although we are unable to directly assess its effects in *Pan*, the fact that the genotype at this position in *Pan* is the same as the human mutator allele, and that the mutator allele in humans is associated with an elevated A-to-T mutation rate, aligns well with the observed acceleration of A-to-T mutations in the human-*Pan* lineage. Together, these results suggest that the *NHEJ1* mutator allele not only explains variation in mutation rates across human populations, particularly in East Asia, but also provides insights into the evolutionary history of mutation spectrum differences between humans and our closest evolutionary relatives. In addition, we examined the effect of heterozygosity versus homozygosity at this site on individual mutation rates (fig. S6). We found that heterozygous genotypes were associated with a significantly elevated mutation rate (Wilcoxon test, *P* = 1.57 × 10^−4^), although no further increase was observed for homozygous genotypes (Wilcoxon test, *P* = 0.66).

One possible mechanism of the *NHEJ1* mutator effect is that the *NHEJ1* mutator allele alters the balance between different modes of DNA end synapsis during NHEJ. In close synapsis, DNA ends are held in a relatively stable and well-aligned configuration by the Ku70-Ku80-XRCC4-LIG4-NHEJ1 complex, which promotes efficient end ligation. In contrast, flexible synapsis maintains DNA ends in a more dynamic and less tightly aligned configuration and involve DNA polymerase μ (Pol μ)–dependent processing (*39*). Because Pol μ is relatively error prone (*40*), a shift from NHEJ1-dependent close synapsis toward more Pol μ–dependent flexible synapsis could increase the probability of single-nucleotide errors during repair. We therefore speculate that reduced *NHEJ1* expression may contribute to the elevated maternal mutation rate and the modest enrichment of A-to-T mutations observed in carriers.

A recent study, which performed a similar GWAS on germline mutation rates based on a larger dataset of over 7000 trios, found no significant SNPs linked to mutation rate variation (*19*). Upon closer examination, we found that the primary difference between their approach and ours was the method of defining the phenotype. In their study, the phenotype was based on mutations phased to either the paternal or maternal origin. In contrast, our analysis used the average number of mutations across the two siblings in each family, which likely provided a more robust and less noisy phenotype. The phasing process used in their study, which relies on nearby heterozygous sites to assign mutations to parental origins, resulted in only 26% of mutations being reliably phased (*19*). This introduces an additional layer of uncertainty in estimating the mutation rate, making the phenotype less accurate and therefore less likely to yield detectable genetic signals in GWAS. To test this hypothesis, we ran an in silico simulation. For each family, we randomly assigned 20% of the total mutations to the maternal side and 80% to the paternal side and then randomly selected 26% of the mutations from the total to assign to the corresponding parental side. In 1000 simulations, the coefficient of variation (CV) for the number of mutations assigned to the maternal side was 0.53, whereas the CV for the paternal side was 0.21 (fig. S7). In contrast, the CV for the mutation counts in our dataset was much lower (0.16). These results suggest that the use of phasing in the previous study could contribute significant noise, particularly for the maternal-origin mutations, which may have hindered their ability to detect significant genetic associations. Furthermore, to simulate the potential impact of this phasing process on GWAS results, we randomly assigned 20% of the mutations as maternal and 80% as paternal for each family in our dataset. We then applied the same 26% phasing rate as in the prior study, phased mutations to their respective parental origins, and used these phased mutations as the phenotype for GWAS analysis. We performed GWAS between the paternal genotype and paternal-origin mutations, as well as between the maternal genotype and maternal-origin mutations. After 100 simulations, no significant SNPs were detected. These results strongly support the idea that the low proportion of phased mutations, particularly when only 26% of mutations can be reliably phased, may lead to a diminished ability to detect causal SNPs in GWAS.

Last, it is important to note that the sample size for our GWAS is smaller than those typically used in previous studies, which may have limited its detection power. Nevertheless, we were still able to identify dozens of significant SNPs. This suggests that additional genetic modifiers of mutation rate and spectrum likely exist within the human population. As more data become available, we anticipate being able to identify even more such modifiers, further enhancing our understanding of the genetic regulation of mutation rates.

## METHODS

### Data collection

The dataset used to analyze the distribution of mutation counts was sourced from Werling *et al.* (*24*) and An *et al.* (*23*), which includes 1902 four-member families. Among these families, we obtained access to whole-genome data for only 1631 quartets, which were used for subsequent GWAS analyses. We used the sample datasets curated by Werling *et al. * (*24*) and An *et al.* (*23*), which excluded any samples with known de novo rare CNVs, de novo loss-of-function mutations, or inherited rare CNVs linked to genetic disorders. In addition, these samples were selected randomly, without regard to parental age, intelligence quotient (IQ), or sex. A total of 6524 genomic Variant Call Format (gVCF) files (1631 quartet families) were downloaded from the SSC for subsequent GWAS analyses (details in table S2). The de novo germline mutation dataset was obtained from Werling *et al. * (*24*) and An *et al.* (*23*), who used a highly accurate detection pipeline and PCR validation (*23*). Data access and analysis were approved by the Institutional Review Boards of Zhejiang University School of Medicine (ID: AF/SC-07, approval no. K2022126).

Our datasets included 1631 quartet families, each comprising a mother, a father, a child diagnosed with autism spectrum disorder (ASD), and an unaffected sibling. The previous literature indicates that mutation rates do not significantly differ between ASD cases and controls (*23*), and our analysis similarly found no significant differences in their mutational spectra (fig. S8). Therefore, this dataset of quartet families is suitable for downstream GWAS analyses of mutation-related traits. After excluding samples with incomplete data in the original files, a total of 3222 samples (*n*_father_ = 1606; *n*_mother_ = 1616) were included in the final GWAS analysis.

### Age correction

Because germline mutation counts are significantly positively correlated with parental age (*20*), we adjusted for paternal and maternal age effects separately. We modeled the relationship between mutation count and parental age using a simple linear regression

$$y=a_{1}x_{\text{father}}+b_{1}\;\text{and}\;y=a_{2}x_{\text{mother}}+b_{2}$$

where *y* is the observed number of de novo mutations in the offspring, $x_{\text{father}}$ and $x_{\text{mother}}$ represent paternal and maternal age, and $a_{1}$ and $a_{2}$ are the regression coefficients. To remove the age effect, we calculated the age-corrected mutation count for each offspring as

$$y_{\text{corrected}}=y_{\text{observed}}-a_{1}\left(x_{\text{father}}-{\bar{x}}_{\text{father}}\right)-a_{2}\left(x_{\text{mother}}-{\bar{x}}_{\text{mother}}\right)$$

where ${\bar{x}}_{\text{father}}$ and ${\bar{x}}_{\text{mother}}$ denote the mean paternal and maternal ages across the cohort. This adjustment removes the linear parental age effect and allows unbiased comparison of mutation counts across individuals for downstream analyses.

### Ancestral inference

Ancestral information for all individuals was inferred using SNVstory (*41*), which relies on the gnomAD model. Developed by Bollas *et al.* (*41*), the gnomAD model is a machine learning–based method that uses a set of 81,398 high-quality Single Nucleotide Variants (SNVs) from the gnomAD v2.1 exome and genome sequencing dataset to infer ancestry. For our participants, we predicted ancestry using the WES mode. Ancestry was classified into five superpopulations at the continental level: African (AFR), South Asian (SAS), European (EUR), East Asian (EAS), and American (AMR). We considered an assignment valid when the probability of belonging to the corresponding population was greater than 80%. We randomly selected 50 fathers and 50 mothers from the GWAS dataset for ancestry analysis, and all were classified as European.

### Genomic data filtering and GWAS analyses

Our analysis began with high-quality gVCF files, which had been generated previously through rigorous filtering of raw sequencing data (*23*). We conducted joint genotyping using GATK (*42*) and subsequently imputed the genomic data using TASSEL (*43*) for all autosomes based on GRCh38. The imputation used the LD KNNi Imputation Plugin, which incorporates the local LD structure and nearest-neighbor relationships to predict missing genotypes. For imputation, we specified up to 30 high-LD sites per locus (-highLDSSites 30), 10 nearest taxa (-knnTaxa 10), and a maximum LD search distance of 100 Mb (-maxLDDistance 100,000,000). Variant-level quality control was performed in PLINK (*44*) by excluding variants with >1% missingness (--geno 0.01), failing Hardy-Weinberg equilibrium at *P* < 1 × 10^−10^ (--hwe 1 × 10^−10^), minor allele count < 100 (--mac 100), or minor allele frequency < 1% (--maf 0.01). Association analysis were performed in rMVP (*45*) using a mixed linear model, with age-adjusted mutation counts as the phenotype. The mixed linear model further included covariates such as age^2^ and five genetically determined principal components to account for potential confounding variables and to control for population structure.

### Definition of independent significant loci and annotation of lead SNP

The significance thresholds for the GWAS analyses were determined using a Bonferroni correction. We first identified independent SNPs by selecting loci with pairwise LD *r*^2^ < 0.1 from the full list of SNPs (determined by LD decay; fig. S9). LD decay was estimated using PLINK by computing pairwise *r*^2^ values between SNPs within 500 Kbp windows. The mean *r*^2^ was summarized across increasing physical distance intervals, and LD decay was visualized as the decline of *r*^2^ with distance. These independent SNPs were then used to estimate the number of effective tests (*N*) in the Bonferroni correction formula (i.e., threshold = 0.05/*N*). As a result, we identified and 120,224 and 122,213 independent loci in the paternal and maternal genomes, respectively, corresponding to genome-wide significance thresholds of 4.15 × 10^−7^ for the paternal dataset and 4.09 × 10^−7^ for the maternal dataset. To identify independent genome-wide significant loci, we applied LD-based clumping using PLINK (*46*). A locus was operationally defined as a genomic region harboring at least one SNP surpassing the primary significance threshold (*P* < 0.05/*N*) and exhibiting LD (*r*^2^ ≥ 0.6) with neighboring SNPs within a 250-kb physical distance. For loci with multiple genome-wide significant SNPs in LD, the SNP with the lowest *P* value was chosen as the lead SNP.

Functional annotation by gene locus and cytogenetic band was performed using ANNOVAR (*47*), based on its precompiled database files hg38_cytoBand.txt and hg38_refGene.txt. The chromatin states of noncoding SNPs were annotated using RegulomeDB (*48*), which integrates ENCODE-based transcription factor (TF) chromatin immunoprecipitation sequencing (ChIP-seq), deoxyribonuclease sequencing (DNase-seq), TF footprints, and quantitative trait locus (QTL) data to identify functionally active regulatory regions. Allele frequency data and genotype data for humans were obtained from gnomAD (*34*).

We also analyzed the association between indel counts and parental genotype but did not identify any significant associations. Therefore, we restricted our analysis to SNVs. We hypothesize that this lack of association is due to the substantially higher variability of indel counts, as reflected by their larger CV (0.59 for indel count versus 0.19 for SNV counts).

### Heritability estimation

We estimated heritability using GWAS data from the paternal and maternal lineages under a variance decomposition framework that explicitly partitions total phenotypic variance into genetic ($V_{g}$) and nongenetic residual ($V_{e}$) components.

We use

$$h^{2}=\frac{\text{Var}\left(\text{additive genetic effect}\right)}{\text{Var}\left(\text{phenotype}\right)}$$

Within the MLM framework

y=Xβ+Zu+ε

where y is the phenotype matrix, Xβ represents fixed effects (e.g., covariates), Z is the identity matrix, $u∼N\left(0,KV_{g}\right)$ (where u is the random genetic effect and K is the genomic relationship matrix), and $\varepsilon ∼N\left(0,IV_{e}\right)$ (where ε represents environmental or other residual effects, and I is the identity matrix).

Then, the variance of y is

$$\text{Var}\left(y\right)=\text{ZK}Z^{T}V_{g}+IV_{e}$$

For each individual $y_{i}$, the variance corresponds to the diagonal element of the *i*th row

$$\text{Var}\left(y_{i}\right)={\left(\text{ZK}Z^{T}\right)}_{\mathrm{ii}}V_{g}+V_{e}$$

Because Z is the identity matrix here, we have

$$\text{Var}\left(y_{i}\right)=K_{\mathrm{ii}}V_{g}+V_{e}$$

and because the diagonal elements of the genomic relationship matrix are 1 ($K_{\mathrm{ii}}$ = 1), we obtain

$$\text{Var}\left(y_{i}\right)=V_{g}+V_{e}$$

Therefore, we estimated heritability using the following formula

$$h^{2}=\frac{V_{g}}{V_{g}+V_{e}}$$

We used the Brent’s method (BRENT) within the REML framework in the rMVP (*45*) software to estimate $V_{g}$ and $V_{e}$.

### Testing for differences in slope and intercept of the linear regressions between maternal age and mutation count

To assess whether the slope and intercept of the linear regressions differ significantly between the two groups (carrier and noncarrier), we performed two linear regression analyses using ordinary least squares (OLS). The first model incorporated maternal age, condition (carrier or noncarrier, coded as 1 or 0), and their interaction term

$$\begin{array}{c} {\text{Mutation number}}_{i}=\beta_{0}+\beta_{1}\times {\text{MotherAgeAtBirth}}_{i}+\beta_{2}\times {\text{Condition}}_{i}\\ +\beta_{3}\times \left({\text{MotherAgeAtBirth}}_{i}\times {\text{Condition}}_{i}\right)+\epsilon_{i} \end{array}$$

where $\epsilon_{i}$ is the residual error, and carrier was treated as the reference level for condition. The coefficient $\beta_{1}$ represents the effect of the maternal age, $\beta_{2}$ represents the mean difference in mutation count between the carrier and the noncarrier when the maternal age is zero, and $\beta_{3}$ represents the difference in the effect of the maternal age between the two groups. The interaction term tests whether the effect of maternal age (i.e., the slope) differs between carriers and noncarriers. If $\beta_{3}$ is significantly different from 0, it indicates that the slopes of the two regressions are significantly different. If $\beta_{3}$ is not significantly different from 0, it means that the slopes are not significantly different.

The second model examined only the main effects of maternal age and condition

$$\begin{array}{c} {\text{Mutation number}}_{i}=\beta_{0}+\beta_{1}\times {\text{MotherAgeAtBirth}}_{i}+\beta_{2}\times \\ {\text{Condition}}_{i}+\epsilon_{i} \end{array}$$

where $\beta_{2}$ reflects the difference in intercepts between the two linear regressions.

In both models, we tested the statistical significance of the regression coefficients using two-sided *t* tests, and *P* values were derived from the corresponding *t* distributions. Effects with *P* values less than 0.05 were considered statistically significant.

### Estimation of the mutation rate contribution from carriers of the mutator allele

When comparing mutation rates in maternal carriers, we treated the paternal side as wild type and did not account for the potential effect of the SNP in fathers on mutation rate. Maternal SNP carrier contribution to germline mutation rate (*C*) is calculated as follows

$$C=\frac{N_{\text{carrier}}-\left(\frac{\alpha }{\alpha +1}\right)\times N_{\text{noncarrier}}-\left(\frac{1}{\alpha +1}\right)\times N_{\text{noncarrier}}}{\left(\frac{1}{\alpha +1}\right)\times N_{\text{noncarrier}}}$$

where *N*_carrier_ and *N*_noncarrier_ represent the mean number of mutations (age-adjusted) in maternal mutation carriers and noncarriers, respectively. α denotes the paternal-to-maternal germline mutation ratio [∼4:1 in Kong *et al.* (*25*)].
