# Supplementary material for: A NHEJ1 mutator allele influencing germline mutation rates sex-specifically in humans
Source: Sci Adv. 2026 Jul 31;12(31):eaed2571. doi: 10.1126/sciadv.aed2571 (PMC13426420; doi:10.1126/sciadv.aed2571)
Supplement: Supplementary file 1 — Figs. S1 to S9 Legends for tables S1 and S2 [file sciadv.aed2571_sm.pdf]

Science Advances
AAAS

Supplementary Materials for

A *NHEJ1* mutator allele influencing germline mutation rates sex-specifically in humans

Kun Wu *et al*.

Corresponding author: Haoxuan Liu, haoxuan@zju.edu.cn

Sci. Adv. **12**, eaed2571 (2026)
DOI: 10.1126/sciadv.aed2571

The PDF file includes:

Figs. S1 to S9
Legends for tables S1 and S2

Other Supplementary Material for this manuscript includes the following:

Tables S1 and S2

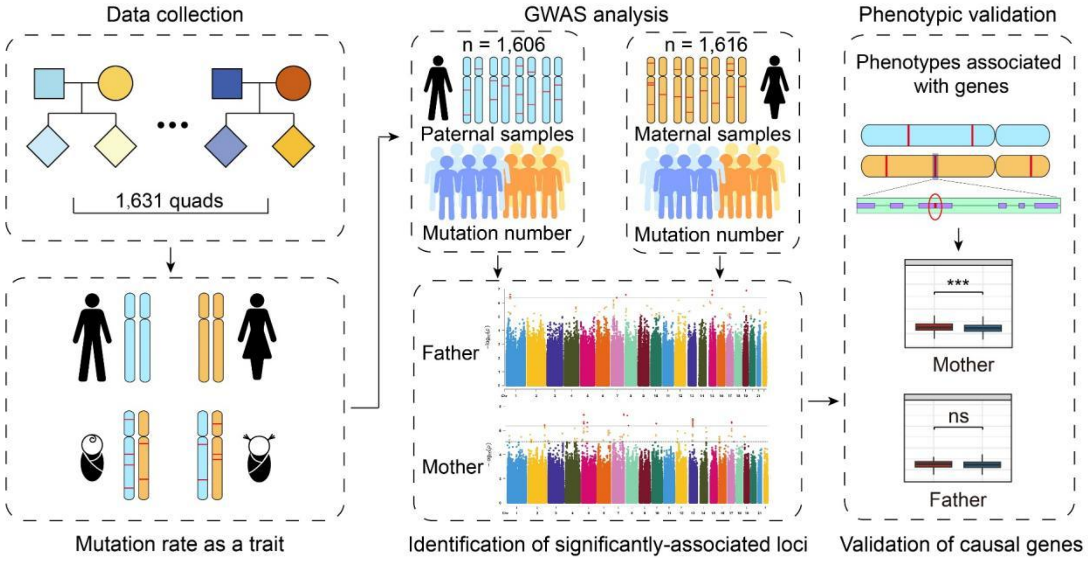

Figure S1. GWAS analysis workflow. We used the number of mutations adjusted for parental age in each family as the phenotype, and conducted separate genome-wide association studies (GWAS) for fathers and mothers. We further assessed the contribution of the detected loci to mutation rates.

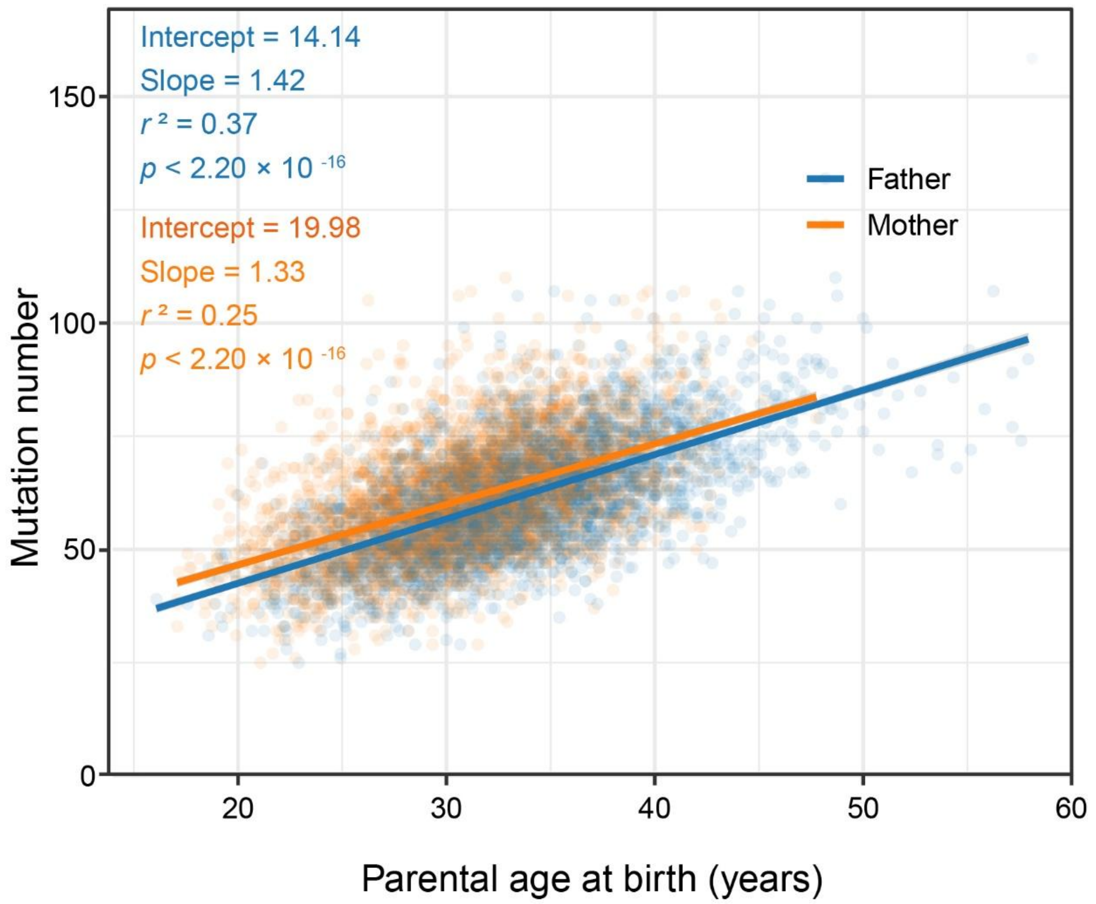

Figure S2. Correlations between paternal and maternal age with mutation number across families. The data were obtained from authorized datasets used for GWAS analysis, including 1,606 paternal samples and 1,616 maternal samples.

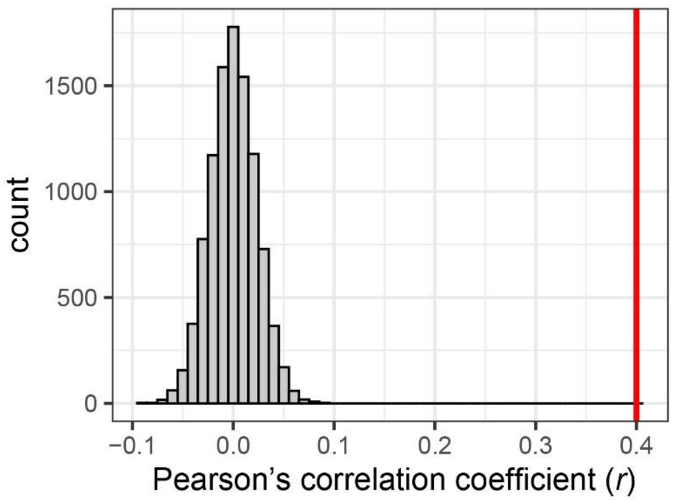

Figure S3. Correlation of mutation counts between siblings after random shuffling. The red line indicates the observed sibling correlation, which differs significantly higher than the null distribution generated by 10,000 random shuffling.

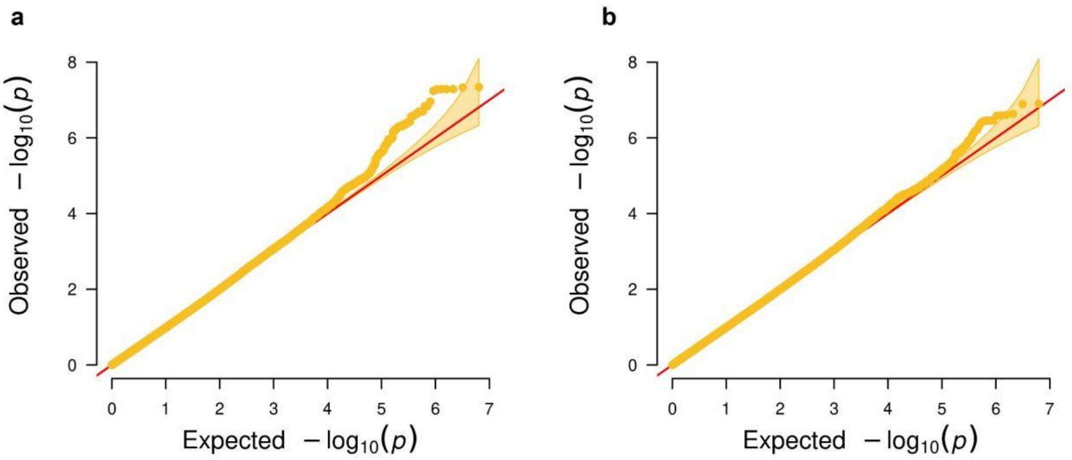

Figure S4. QQ (Quantile–quantile) plots for maternal (a) and paternal (b) datasets.

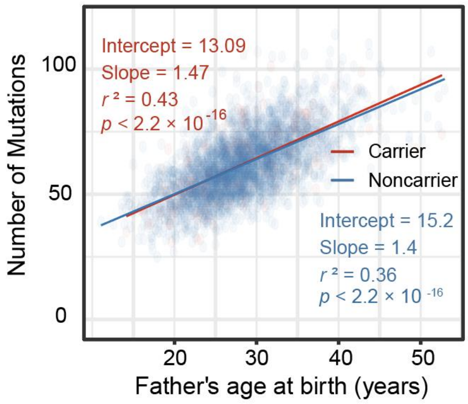

Figure S5. The correlation between age and the number of mutations in individuals carrying and not carrying the lead SNP in the paternal dataset.

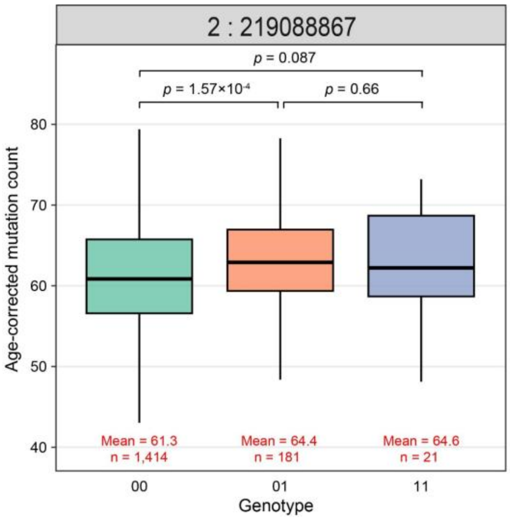

Figure S6. Age-corrected mutation counts for heterozygous and homozygous genotypes in the maternal dataset at the lead SNP in *NHEJ1*. The genotype of 00, 01, and 11 represent noncarriers, heterozygous carriers, and homozygous carriers, respectively and n represents the number of samples with this genotype. The *p* value was calculated using the Wilcoxon test.

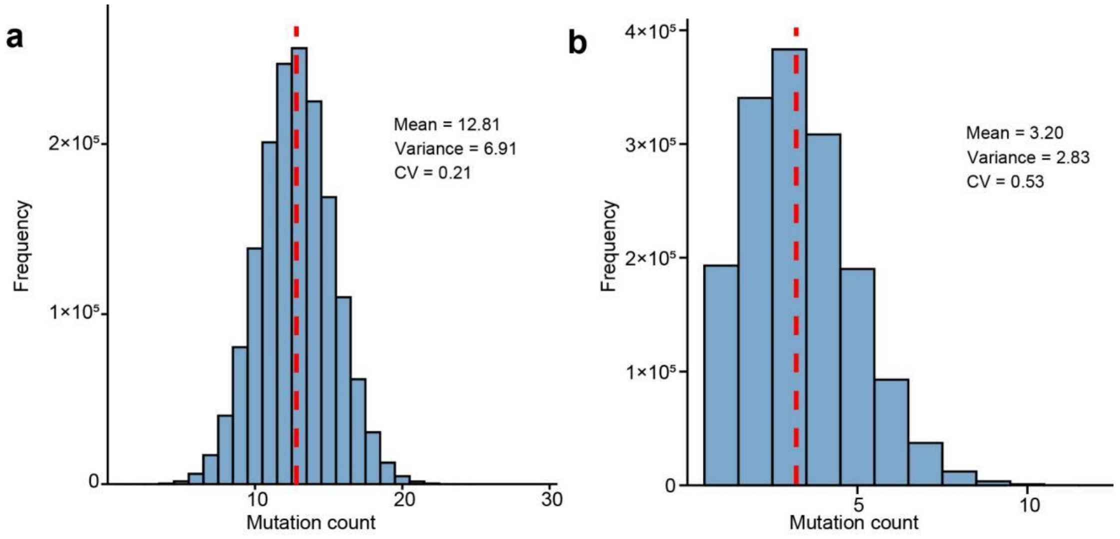

Figure S7. Computational simulation of phenotype data after the incorporation of the phasing process. Figure shows the distributions of mutation counts phased to paternal (**a**) and maternal (**b**) origin.

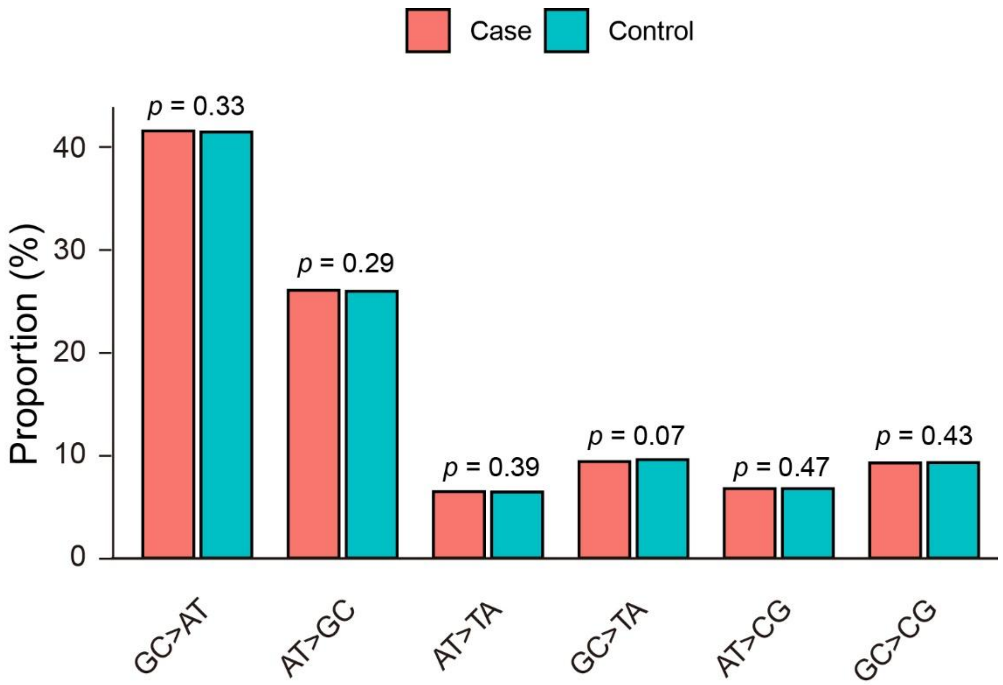

Figure S8. Comparison of mutation spectra between autism cases and their sibling controls. The mutation profiles show no significant differences, which is consistent with previous analysis (*24*). The *p* values shown above were calculated using a permutation test.

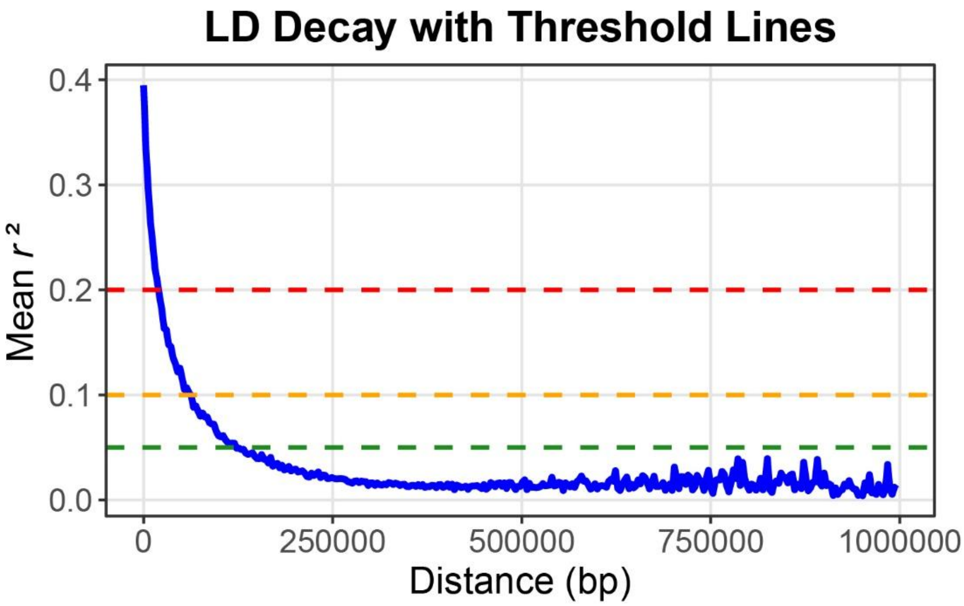

Figure S9. Linkage disequilibrium (LD) decays with increasing physical distance between variants.

Table S1. RegulomeDB chromatin state annotations for the lead and suggestively associated SNPs within *NHEJ1*.

Table S2. Summary of parental mutation information (before age correction, data from An *et al.* 2018 (*23*)).
